# Development and validation of a convolutional neural network to identify blepharoptosis

**DOI:** 10.1038/s41598-023-44686-3

**Published:** 2023-10-16

**Authors:** Cristina Abascal Azanza, Jesús Barrio-Barrio, Jaime Ramos Cejudo, Bosco Ybarra Arróspide, Martín H. Devoto

**Affiliations:** 1 Department of Ophthalmology, Navarra Institute for Health Research (IdiSNA), Clínica Universidad de Navarra, Av. de Pío XII, 36, 31008 Pamplona, Navarra Spain; 2 Faculty of Medicine, Universidad de Navarra, Pamplona, Spain; 3 Grossman School of Medicine, New York University (NYU, New York, USA; 4 Imagine Apps, Madrid, Spain; 5 Consultores Oftalmologicos, Buenos Aires, Argentina

**Keywords:** Eye manifestations, Computational models

## Abstract

Blepharoptosis is a recognized cause of reversible vision loss and a non-specific indicator of neurological issues, occasionally heralding life-threatening conditions. Currently, diagnosis relies on human expertise and eyelid examination, with most existing Artificial Intelligence algorithms focusing on eyelid positioning under specialized settings. This study introduces a deep learning model with convolutional neural networks to detect blepharoptosis in more realistic conditions. Our model was trained and tested using high quality periocular images from patients with blepharoptosis as well as those with other eyelid conditions. The model achieved an area under the receiver operating characteristic curve of 0.918. For validation, we compared the model's performance against nine medical experts—oculoplastic surgeons, general ophthalmologists, and general practitioners—with varied expertise. When tested on a new dataset with varied image quality, the model's performance remained statistically comparable to that of human graders. Our findings underscore the potential to enhance telemedicine services for blepharoptosis detection.

## Introduction

It is widely recognized that AI has found extensive application in ophthalmology, particularly in the field of retinal disease diagnosis. This is evidenced by over 2000 published studies found on PubMed that combine the terms 'retina' and 'artificial intelligence.' The prevalence of AI applications in retinal diseases can, in part, be attributed to the availability of large, high-quality image datasets that facilitate algorithmic training. Conversely, the field of oculoplastics has been slower to adopt AI, with only 14 published studies on PubMed combining the terms 'oculoplastics' and 'artificial intelligence' as of a search conducted on September 2, 2023. Challenges in oculoplastics include the variability in clinical presentations and the lack of standardized, large-scale datasets.

Despite these obstacles, experience in applying AI to oculoplastics is on the raise and it is anticipated that further research in this specialized area will gain momentum in the near future^1,2^. Existing CNNs feature filters capable of identifying low-level structural elements such as color, contrast, and edge detection^3^. These filters enable CNNs to undergo 'end-to-end' training, eliminating the need for pre-processed input and requiring only raw image data.

Oculoplastics is an area particularly suited for CNNs and tele-ophthalmology, as visual information and unstructured data can be easily acquired using non-specialized equipment. Within the field of oculoplastics, blepharoptosis (i.e., drooping of the upper eyelid) stands as a clinically significant condition, often serving as the initial or sole manifestation of severe disorders^4^. For instance, blepharoptosis can manifest in the context of Horner syndrome (HS), which may occasionally signify life-threatening conditions such as neuroblastoma in children or carotid artery dissection and aneurysms in adults^5–7^. To date, AI-based diagnosis of blepharoptosis has largely relied on parameters established by routine clinical practice, including the measurement of Margin to Reflex Distance 1 (MRD1) and palpebral fissure height^8^. These approaches primarily employ digital image processing techniques to identify relevant clinical parameters. Recently, however, there has been a shift towards the application of deep learning DL, specifically CNNs, for diagnosing blepharoptosis. A study by Hung et al. reported the successful use of AI to accurately diagnose blepharoptosis from clinical photographs, without the need for external reference markers or user input, using single-eye images from an Asian ethnic background^9,10^.

In this study, we offer a holistic approach to the diagnosis of blepharoptosis using CNNs. Specifically, we utilize images of both eyes, including the eyebrows, and train a single CNN model end-to-end using only pixel values and disorder labels as inputs. Our control group consists of healthy individuals as well as patients with other types of eyelid conditions. This provides a diagnostic framework more reflective of what we might encounter in a routine oculoplastic consultation. It should be emphasized that our research serves as a proof of concept, laying the groundwork for future investigations in this area.

## Methods

Informed consent was obtained from all subjects and/or their legal guardians for both participation in the study and the publication of identifying images in an online open-access journal. The clinical study received approval from the Research Ethics Committee/Institutional Review Board of Navarra University Hospital and was conducted in accordance with the principles outlined in the Declaration of Helsinki.

### Data collection and preprocessing

This study utilized original photographs collected from a tertiary oculoplastic clinic over a 25-year period, spanning from 1993 to 2018. The dataset includes 250,534 periocular and facial images from 10,555 patients. All images were rigorously graded by a single expert in line with the patients' conditions. It is worth noting that the photographs were captured using various cameras and resolutions over the years.

**Figure 1:**
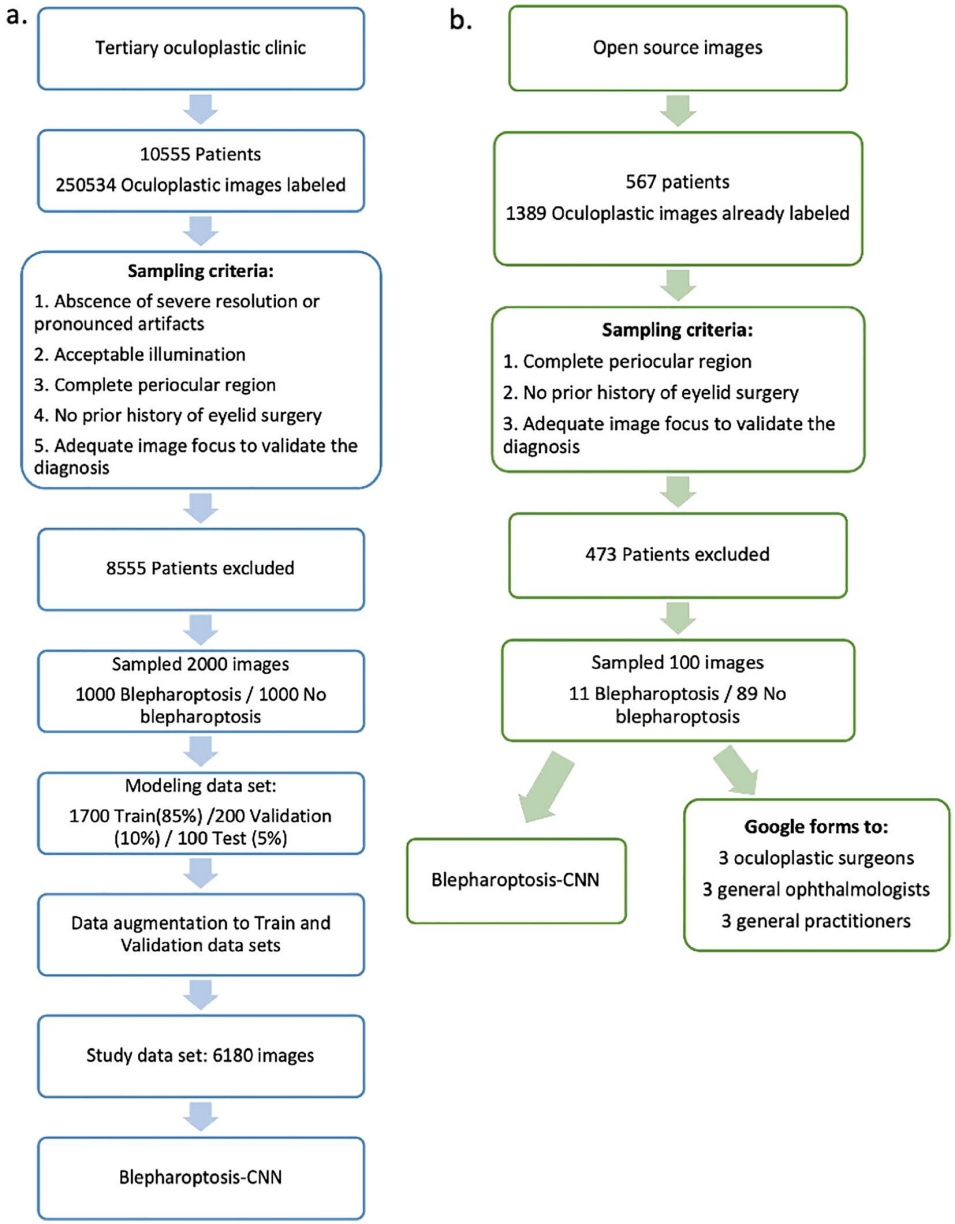
Workflow for the development of the Blepharoptosis-CNN (**a**) and the comparative study (**b**)**.**

From the entire dataset, 2000 periocular images were chosen, comprising 1000 patients diagnosed with blepharoptosis and another 1000 without the condition. For every patient, one frontal photograph was incorporated. Each image added to the database was binary- labeled either as "blepharoptosis" or "no blepharoptosis." It's noteworthy that patients categorized under "no blepharoptosis" were not necessarily devoid of any pathologies or disorders; they may have exhibited medical conditions distinct from blepharoptosis. Initial image quality control was overseen by a member of the research team based on the following criteria:

1. Absence of severe resolution diminution or pronounced artifacts such as hair or ruler interferences.
2. Acceptable illumination, ensuring the image was neither excessively dark nor overly bright.
3. Image composition that encompassed the complete periocular region.
4. No prior history of eyelid surgery.
5. Adequate image focus to validate the diagnosis (Fig. 1).

The ground truth for blepharoptosis was established via consensus among three oculoplastic surgeons using a voting system. Initially, two of the three labelers independently reviewed all 2000 photographs in the primary labeling phase. For images where a consensus could not be reached, adjudication was performed by a third, independent oculoplastic surgeon.

### Model architecture and training

To develop our state-of-the-art DL model, Blepharoptosis-CNN, we used labeled training images. We implemented the model using the Keras API within TensorFlow, basing it on the VGG-16 architecture. We made several key modifications to the VGG-16 architecture to meet our specific needs. Initially, we omitted one convolutional layer and its associated max-pooling layer, reducing the count from five to four, based on observed performance improvements. We then replaced the terminal dense layer with a dropout layer with a rate of 0.5 to facilitate model regularization. This was followed by additional fully connected layers for feature integration. Finally, a sigmoid activation function was integrated into the output layer to fulfill the binary classification objective of our study.

To address the prevalent issue of overfitting, especially common in deep CNNs, we employed data augmentation and dropout techniques within our image classification architecture. Overfitting arises when a model, due to its high capacity, captures not only the overarching features but also the subtle irregularities and noise in the training data, thereby affecting its generalization performance on new, unseen data. Such overfitting is often exacerbated when training on limited datasets with models that have numerous parameters^11^. To fine-tune our training process, we regulated the number of complete passes through the training dataset using a parameter known as 'epochs,' which is defined as one complete forward and backward pass of the entire dataset through the neural network.

Our analysis employed a binary classification approach, categorizing selected images into two groups: those showing patients with 'blepharoptosis' and those without the condition. The images were preprocessed to a resolution of 128 × 64 pixels using software from Sketch B.V. and centered on the periocular area, including the eyebrows, for targeted analysis. The dataset was randomly divided into distinct subsets: 85% for training, 10% for validation, and the remaining 5% for testing. The test set was reserved for evaluating the final model's performance and was not used during the model development stage.

**Figure 2:**
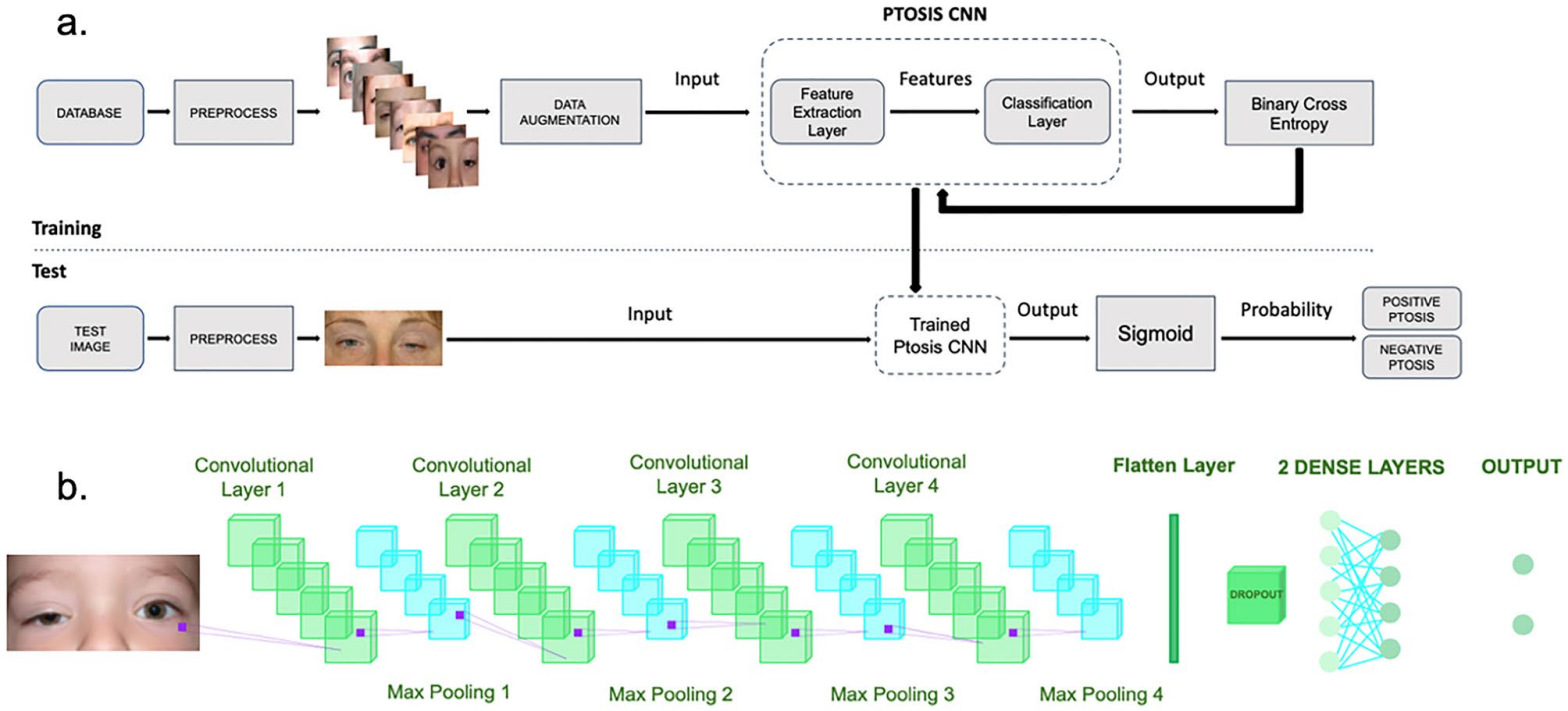
Blepharoptosis-CNN, (**a**). The framework of the DL model for detecting possible blepharoptosis, (**b**). The structure of Blepharoptosis-CNN. The VGG-16 architecture provided the fundamental framework for constructing the model of Blepharoptosis-CNN.

Given the limited size of our dataset and to mitigate the risk of overfitting, we implemented data augmentation techniques. These methods artificially expanded our training dataset, thus enhancing the model's generalization capabilities. Specifically, training and validation images underwent random transformations such as horizontal flips, rotations, and zoom adjustments. Data augmentation was exclusively applied to the training and validation sets, ensuring that the test set remained unmodified for unbiased performance assessment (Fig. 2).

Metrics were calculated and graphs were generated using Python (version 3.7.12, Python Software Foundation). A variety of packages, including Mmatplotlib, Sscikit-Llearn, Nnumpy, and Ppandas, were employed for these tasks.

### Comparative study

To assess the effectiveness of the CNN-Blepharoptosis in genuine healthcare settings, a comparative study was conducted juxtaposing its performance against human graders utilizing periocular images from online databases previously unseen by the Blepharoptosis- CNN. We collated a dataset of 100 periocular images, originating from various publicly accessible databases including Google Images and an open image database from "https://www.realself.com." The image dataset comprised both diagnosed blepharoptosis cases and control subjects. We targeted front-facing images that offered a comprehensive view of the periocular region, encompassing both eyes and eyebrows. No constraints were applied to image quality, thereby capturing the diverse spectrum of clinical scenarios, including patient-generated selfies. Two board-certified oculoplastic surgeons, boasting 20 and 3 years of experience respectively, independently annotated the selected images. Wherein consensus was unattainable, a third board-certified oculoplastic surgeon served as an adjudicator.

Our human grader panel consisted of nine medical professionals, grouped into three experience-based categories: experts with over 20 years of experience, competent individuals with 5–20 years, and novices with less than 5 years. All selected images were downscaled to a 128 × 64-pixel resolution, aligning with the input dimensions stipulated by the CNN-Blepharoptosis training dataset, utilizing Sketch software for the downsizing procedure. Importantly, we did not alter the brightness or contrast of the images.

Graders were presented images through a structured Google Forms interface, which required a binary response—either ¨blepharoptosis¨ or ¨no blepharoptosis¨. The term "blepharoptosis" was clinically defined based on the subjective identification of drooping in one or both upper eyelids, consistent with the criteria that would instigate further clinical evaluation in real-world settings. All participants interacted with the identical test document, devoid of any supplemental information, to ensure the objectivity and repeatability of the evaluation process.

Through this meticulously crafted study design, we aimed to capture the nuances of practical applicability and real-world performance of CNN-Blepharoptosis.

### Statistical analysis

The learning performance of the AI algorithm was assessed using the conventional metric of ROC AUC (Area Under the Receiver Operating Characteristic Curve) during both training and field testing on the study dataset. Throughput was evaluated using standard measures, including sensitivity, specificity, and the Youden Index score. Model performance metrics were calculated based on the counts of true positive samples (TP), false positive samples (FP), true negative samples (TN), and false negative samples (FN).

$$Accuracy=\frac{TP+TN}{TP+FP+TN+FN}$$

$$Sensitivity=\frac{TP}{TP+FN}$$

$$Specificity=\frac{TN}{FP+TN}$$

$$Youden Index=Sensitivity \left(\%\right)+Specificity \left(\%\right)-100$$

ROC AUC = Area under the Receiver Operating Characteristic curve.

Where statistical significance was assessed, *p* values were calculated using Fisher’s exact test, Kruskal Wallis and Chi-squared with a *p*-value < 0.05 considered significant.

## Results

Utilizing images gathered from oculoplastic clinic evaluations and after applying data augmentation techniques, this study included a total of 6180 periocular images. The dataset comprised 1000 patients diagnosed with blepharoptosis and 1000 patients without the condition. Of these patients, 38.7% were men and 61.3% were women, with a mean age of 57 ± Q1 41.0, Q3 64.2 years. No significant differences in ethnicity were observed (*p* = 0.250; Fisher's exact test). However, significant differences were identified in terms of age (*p* = 0.002; Kruskal–Wallis test), sex, and laterality (*p* = 0.031 and *p* = 0.001, respectively; Chi-squared test) between the two groups. The demographic characteristics of the cohort are summarized in Table 1.

**Table 1:** Summary of dataset.

n		Overall	No Ptosis	Ptosis	*P* < *.05*	Test
		2000	1000	1000		
Age, median [Q1,Q3]		57.0 [41.0, 64.2]	57.0 [45.0, 63.0]	54.0 [25.0, 65.0]	0.002	Kruskal–Wallis
Sex, n (%)	Female	1226 (61.3)	637 (63.7)	589 (58.9)	0.031	Chi-squared
	Male	774 (38.7)	363 (36.3)	411 (41.1)		
Ethenic, n (%)	Caucasian	1997 (99.9)	997 (99.7)	1000 (100.0)	0.250	Fisher´s exact
	Asian	3 (0.1)	3 (0.3)			
Laterality, n (%)	Bilateral	1016 (50.8)	392 (39.2)	624 (62.4)	< 0.001	Chi-squared
	Unilateral	984 (49.2)	608 (60.8)	376 (37.6)		

**Figure 3:**
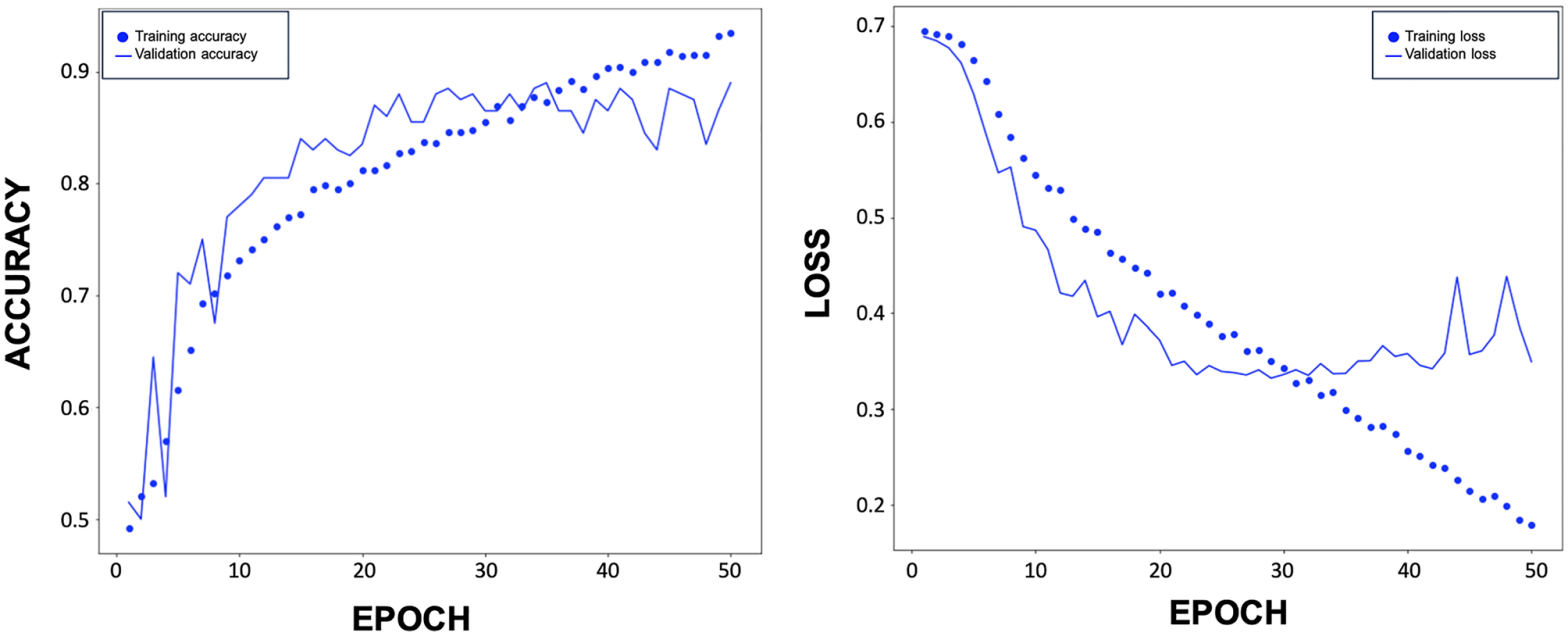
Training curve for the Ptosis-CNN. Blue dot, Training Learning Curve: Learning curve calculated from the training dataset that gives an idea of how well the model is learning. Blue dash, Validation Learning Curve: Learning curve calculated from the hold-out validation dataset that gives an idea of how well the model is generalizing. After training for 20 epochs, our DL model showed no improvement in both accuracy and loss function.

**Figure 4:**
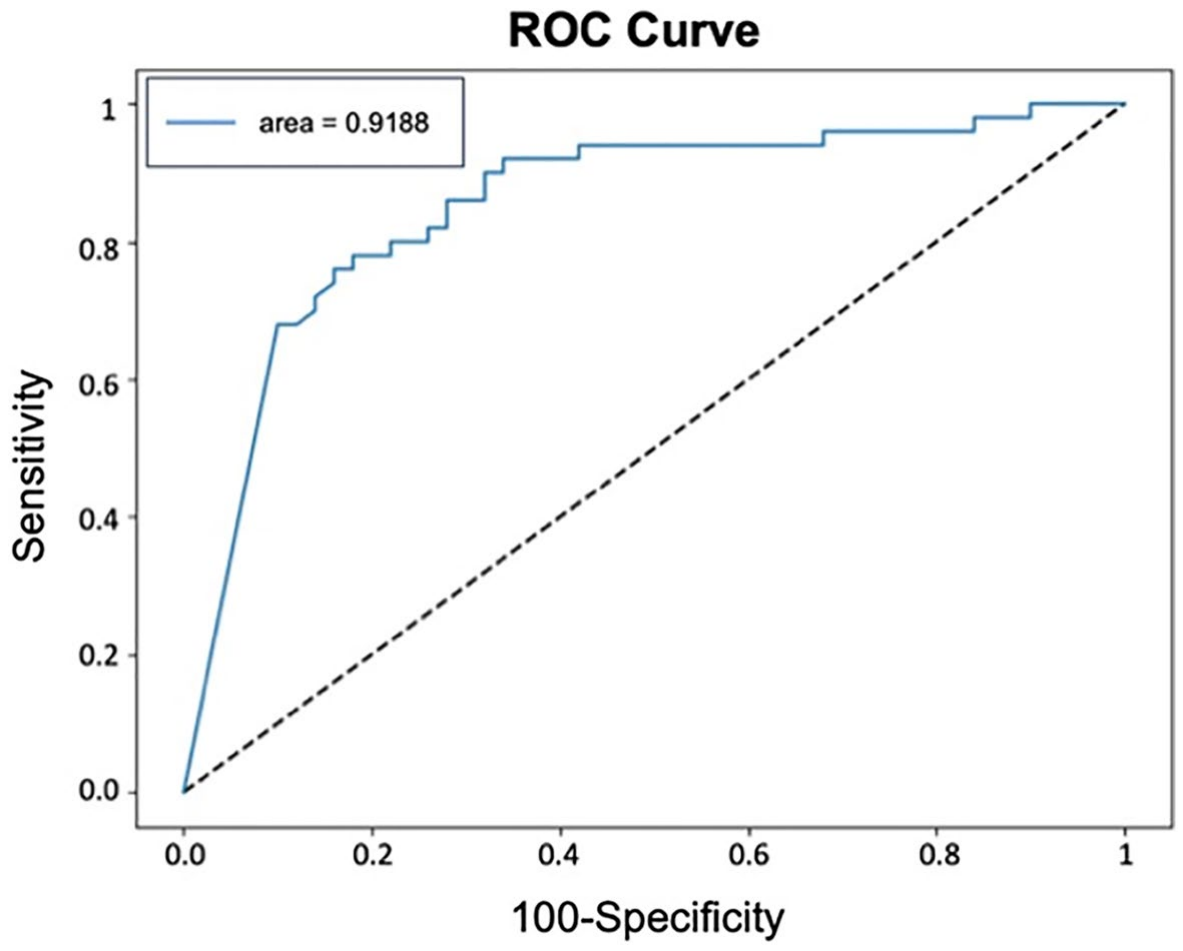
ROC curve. Performance of the CNN in detection of blepharoptosis in the testing set. The area under the curve (AUC) is 0.918.

After training the DL model for 20 epochs (as shown in Fig. 3), the model achieved an AUC of 0.918 for distinguishing between patients with and without blepharoptosis (Fig. 4). The model exhibited a sensitivity of 98% and a specificity of 88%. The Youden Index score for our model stood at 0.860.

In the comparative study aimed at assessing the trained AI algorithm's efficacy in real-world healthcare settings, we included 100 periocular images of unique patients—11 with blepharoptosis and 89 without. Characterized by varying image quality, the achieved AUC for this dataset was 0.700. The model exhibited a sensitivity of 54% and a specificity of 85% (Table 2). Upon juxtaposition of this performance against the highest and lowest AUCs of human graders, the observed differences (0.142 and 0.0618, respectively) were found to be statistically non-significant (*p* = 0.6328 and *p* = 0.2220, respectively). However, the performance discrepancy between the best and worst human graders, quantified as an AUC difference of 0.204, was statistically significant (*p* = 0.0352) (Table 3).

**Table 2:** Summary of the Area Under the ROCs, Sensitivity—Specificity Balance, Youden Index Score and *P* Value of the Blepharoptosis-CNN and Human Graders in the comparative study.

		%(95%CI)			
Model comparison	AUC (95% CI)	Sensitivity	Specificity	Youden index score	*P* < .05
Blepharoptosis-CNN	0.700 (0.600–0.787)	54.5 (23.3–83.2)	85.3 (76.3–91.9)	0.399	0.0339
Oculoplastic surgeons					
Expert	0.842 (0.755–0.907)	81.8 (48.2–97.7)	86.5 (77.6–92.8)	0.683	0.0001
Competent	0.836 (0.749–0.903)	81.8 (48.2–97.7)	85.3 (76.3–91.9)	0.671	0.0001
Novice	0.825 (0.736–0.894)	81.8 (48.2–97.7)	83.1 (73.7–90.2)	0.649	0.0001
General ophthalmologists					
Expert	0.836 (0.748–0.902)	72.7 (39.0–93.9)	94.3 (87.3–98.1)	0.671	0.0001
Competent	0.768 (0.673–0.846)	63.6 (30.7–89.0)	89.8 (81.6–95.2)	0.535	0.0006
Novice	0.711 (o.612–0.797)	54.5 (23.3–83.2)	87.6 (78.9–93.6)	0.421	0.0089
General practitioners					
Expert	0.761 (0.666–0.841)	54.5 (23.3–83.2)	97.7 (92.1–99.7)	0.523	0.0010
Competent	0.656 (0.554–0.748)	72.7 (39.0–93.9)	58.4 (47.4–68.7)	0.311	0.0382
Novice	0.638 (0.536–0.732)	54.5 (23.3–83.2)	73.0 (62.5–81.8)	0.275	0.0935

**Table 3:** Differences in the AUC Among the Blepharoptosis-CNN, the best and the worst Human Graders.

Model comparison	Differences between AUCs	95% CI	*P* < .05
Blepharoptosis-CNN versus Expert Oculoplastic Surgeon	0.142	0.0859–0.370	0.2220
Blepharoptosis-CNN versus Novice General Practitioner	0.061	0.192–0.315	0.6328
Expert Oculoplastic Surgeon versus Novice General Practitioner	0.204	0.0141–0.393	0.0352

**Figure 5:**
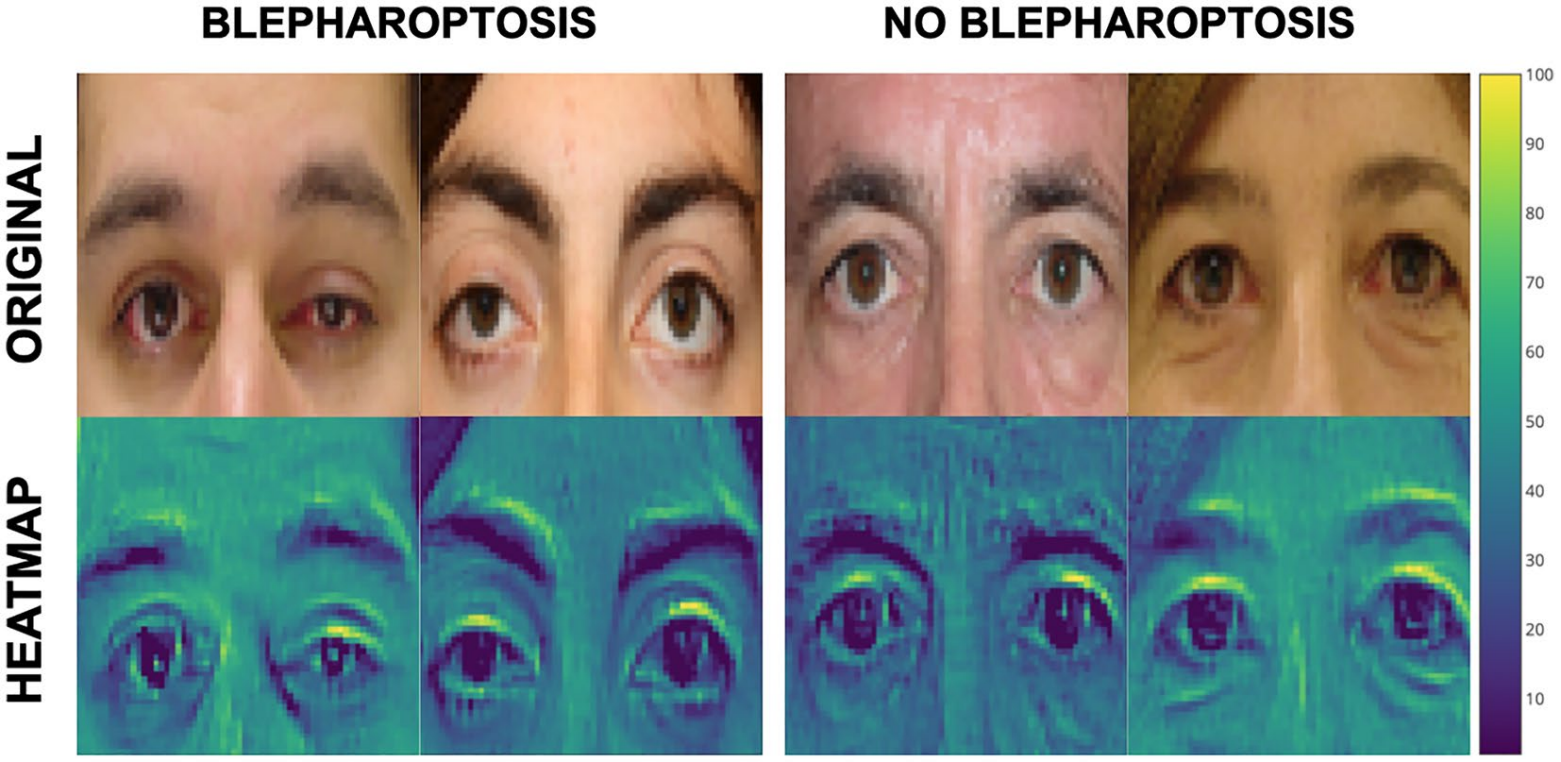
Visualization of prediction imaging features.

In our analysis of the contributions from different regions of the periocular images to Blepharoptosis-CNN's predictions, we found that activation primarily occurred in the upper lid margin and upper brow area (Fig. 5). These regions are highlighted in heat maps, which serve to identify the most diagnostically significant areas of the periocular images. These heat maps, often referred to as saliency maps, delineate the unique characteristics—such as pixels and resolution—that the network focuses on for its predictions within the context of visual processing^12^.

The heat map was mapped to the original image to visualize the importance of each region.

in Blepharoptosis prediction. The Blepharoptosis-CNN was able to identify characteristic areas (superior margin of the eyelid and superior area of eyebrows, yellow arrow) in periocular photographs, which are presented as a heat map. The yellow color represents the heatest area for extraction of features for detecting blepharoptosis.

## Discussion

The present study introduces a holistic method for differentiating between patients with and without blepharoptosis in actual clinical settings. In instances involving high-definition images, the CNN displayed exceptional diagnostic accuracy, evidenced by an AUC of 0.918. Even in the case of low-quality images, the system maintained a respectable performance, registering an AUC of 0.700.

When compared to analogous studies with high-quality images, our model exhibits comparable performance metrics. For instance, J. Hung et al. reported in their article 'A Deep Learning Approach to Identify Blepharoptosis by Convolutional Neural Networks' that their top-performing CNN model attained a sensitivity of 90.1% and a specificity of 82.4%^9^. In a subsequent investigation, Hung et al. deployed an AI model based on the VGG-16 architecture and utilized more extensive and diverse datasets to diagnose blepharoptosis accurately. Transfer learning was applied by importing pretrained weights from ImageNet, resulting in their CNN model achieving a sensitivity of 92% and a specificity of 88%^10^. In contrast, our modified model of VGG-16 showed superior performance in sensitivity, achieving a rate of 98%, while maintaining the same specificity rate of 88%. Therefore, this supports the idea that the benefit of transfer learning is limited when working with this particular type of eye images.

In these research studies focused on an Asian population, Hung et al. trained their DL-CNN models using images that displayed only one eye and excluded the eyebrow^9,10^.

However, it is worth noting that determining in advance which anatomical features will be most useful for diagnosis is challenging, as CNN models are self-learning^12^. Consequently, the clinical images used in our study are meticulously centered on the periocular domain, including the eyebrows within the frame. The frontalis muscle, apart from its role in elevating the forehead and eyebrows, also serves an ancillary function in lifting the upper eyelid, providing an additional elevation of 3–5 mm. Noteworthy findings from our study's heatmap analyses indicate pronounced activations primarily in the superior eyelid margin and the upper eyebrow region within the images generated by the Blepharoptosis-CNN. These revelations could serve as a pivotal reference for future iterations of Blepharoptosis-CNN implementations.

Another distinct facet of our approach resides in the integration of both eyes and eyebrows within the clinical photographs, an innovative paradigm in the context of CNN utilization. Traditional AI algorithms for blepharoptosis have primarily focused on quantifying eyelid position in images that display a single eye^13–20^. Notably, there is a dearth of antecedent reports scrutinizing the efficacy of CNNs when applied to images encompassing both ocular elements simultaneously. Our deployed CNN exhibits the capacity to accurately classify instances of blepharoptosis, irrespective of the condition manifesting unilaterally (in one eye) or bilaterally (in both eyes). This crucial capability mirrors the inherent nature of clinical evaluation, a paradigm where gauging each eye in isolation may inadvertently distort the accurate depiction of blepharoptosis, potentially culminating in misdiagnosis or susceptibility to instances of misrepresentation, as might be encountered in interactions with medical insurers. This underscores the relevance of adhering to Hering's Law of equal innervation, positing symmetrical innervation of ocular and eyelid muscles. In scenarios involving marked ptosis in one eye, the principle of equal innervation necessitates the elevation of the contralateral eyelid^21^. Should the ptotic eyelid be elevated, the opposing eyelid is inclined to descend due to the reciprocal relationship in stimulus response. This physiological framework further accentuates the necessity of adopting a holistic visual perspective for accurate assessment.

To date, studies have trained their CNNs with images of blepharoptosis patients and healthy patients [^9^][^10^][^14–16^]^9,10,13–15^. However, in real clinical settings a physician evaluates patients with different types of pathologies and disorders that can potentially influence the interpretation of facial features^22^. Notably, our study embraces a more expansive patient cohort, encompassing individuals with blepharoptosis, healthy subjects, and remarkably, those presenting with diverse eyelid pathologies other than blepharoptosis (e.g., thyroid eye disease, eyelid tumor, ectropion…). This holistic inclusion stands as a significant departure from prior reports, wherein such a comprehensive patient spectrum had not been previously explored. This strategic inclusion serves as a pivotal facet of our evaluation strategy, as a substantial proportion of patients seeking consultation with specialized ophthalmologists are inclined to manifest diverse palpebral or orbital pathologies. Furthermore, it merits emphasis that our study design distinctly excludes patients who have undergone prior oculoplastic interventions within the non-blepharoptosis group. We posit that the distinctive contour of the eyelid ensuing from blepharoptosis surgery may not faithfully mirror the native eyelid topography in a healthy subject. This premise is discernible in the work by Tabuchi et al.^13^ wherein automated ptosis diagnosis was executed using a pretrained MobileNetV2 CNN applied to images captured via an iPad Mini. An incisive examination of Fig. 2 of their publication unveils an instance (labeled “b”) as a normal eyelid. However, discernible alterations in eyelid skin hue (manifesting as redness) and a distinct tapering form at the apex of the upper eyelid margin are evident. The latter phenomenon is frequently an aftermath of inadvertent folding of the tarsal plate during suture placement, as is often observed post-surgery.

Since the onset of the COVID-19 pandemic, telemedicine has become an integral component of oculoplastic service delivery, and its continued use is anticipated^23^. This mode of healthcare provision offers a streamlined, efficient approach for the preliminary assessment of patients suspected to have blepharoptosis, whether in the context of potential systemic or neurological disorders or for expedited referrals to oculoplastic specialists. It is precisely this growing reliance on telemedicine for oculoplastic evaluations that underscores the critical importance of conducting validation studies for diagnostic algorithms in real-world clinical settings.

The comparative study conducted by Hung et al. yielded promising results, showing that their CNN model outperformed non-ophthalmic physicians in identifying both true and pseudoptosis cases of referable blepharoptosis. Their CNN model attained an AUC of 0.90, compared to a mean AUC of 0.77 for the non-ophthalmic physician group when utilizing high-quality images^10^. From an AUC standpoint, our algorithm demonstrated performance comparable to that of human graders. Nonetheless, there is a notable difference between the AUC of 0.918 achieved with the high-quality image test set and the AUC of 0.700 attained with the low-quality image test set, as observed in the comparative study. Our intention with this comparative study was to push the limits of our Blepharoptosis-CNN and to stimulate discussion regarding what the achievements of AI signify when subjected to controlled studies versus their real-world applicability in uncontrolled conditions.

It's essential to highlight the differences in image quality between the two studies. While Hung et al. relied on high-quality images with optimal focus, brightness, and minimal artifacts, our study incorporated the kinds of images one might typically encounter in a standard telemedicine consultation. Real-world healthcare settings often present clinicians with challenges like variable lighting and inconsistent capture distances. Our intentional choice to include such images adds an extra layer of robustness to our findings, emphasizing the algorithm's ability to function effectively under less-than-optimal conditions.

Despite the absence of high-caliber images, our model exhibited remarkable resilience across a range of palpebral apertures and image resolutions. Looking ahead, broader adoption of telemedicine will require further advancements in digital infrastructure and clinical examination capabilities. For ophthalmologists, telemedicine also has the potential to streamline processes, possibly serving as a convenient alternative to manual MRD1 measurements and visual field tests for insurance approvals.

This study presents several limitations that warrant further discussion. Specifically, our Blepharoptosis-CNN was trained exclusively on high-quality images, which has affected its AUC performance when applied to poor-quality images commonly found in non-specialized clinical settings^24^. When a CNN is subjected to a test set featuring images of lower resolution than those used in the training and validation datasets, several challenges may arise. First, there is the issue of dimensional incompatibility, as CNNs are engineered to accept input of a specific size; test set images with fewer pixels must therefore be resized to align with the network's expected input dimensions. Second, this resizing process may result in a critical loss of detail, particularly important for tasks such as classification or object detection. Lastly, reduced performance is a concern; given that the CNN was trained on high-resolution images, its ability to generalize effectively to lower-resolution images may be compromised, potentially impacting accuracy, sensitivity, or other key performance metrics. In summary, the application of a high-resolution-trained CNN to a lower-resolution test set introduces a range of complications, from dimensional mismatch to performance degradation, underscoring the necessity to incorporate images of varying quality and size in both training and validation stages. Consequently, we believe it is crucial to develop extensive oculoplastic databases akin to those available in other fields of ophthalmology, such as retinal studies. The necessity for more extensive databases specifically tailored for oculoplastic studies suggests a roadmap for improving the diagnostic capabilities and generalization of AI models.

Second, our dataset exhibited substantial variations in terms of sex, age, and laterality, which may have influenced the Blepharoptosis-CNN's ability to accurately classify periocular images. Third, the dataset employed for training the CNN was overwhelmingly comprised of images from patients of Caucasian descent, making up nearly 99% of the sample. It is plausible that the model's sensitivity and specificity could decline when applied to diverse racial groups, as the pixel-intensity relationships tied to blepharoptosis could differ across ethnicities. Thus, the applicability of our CNN model is largely restricted to Caucasian populations. Fourth, there is a need for advancements in medical image processing techniques to enhance accuracy, computational efficiency, and overall performance. Although the CNN model employed in this study has shown effectiveness in image classification tasks, it primarily serves as a proof of concept. Future research targeting the detection of blepharoptosis in the context of other ocular conditions should explore the incorporation of hybrid techniques and more advanced methods for hyperparameter optimization.

## Conclusions

This study developed Blepharoptosis-CNN, a DL model demonstrating high diagnostic accuracy in controlled clinical settings. The model's performance compared favorably with human graders, thus validating its applicability in healthcare settings.
